# Ubiquitin conjugating enzymes participate in polyglutamine protein aggregation

**DOI:** 10.1186/1471-2121-8-32

**Published:** 2007-07-30

**Authors:** Rebecca A Howard, Pratima Sharma, Connie Hajjar, Kim A Caldwell, Guy A Caldwell, Rusla du Breuil, Rhonda Moore, Lynn Boyd

**Affiliations:** 1 Department of Biological Sciences, University of Alabama in Huntsville, Huntsville, AL 35899, USA; 2 Department of Biological Sciences, University of Alabama, Tuscaloosa, AL 35487, USA; 3 Open Biosystems, Inc., Huntsville, AL 35806, USA

## Abstract

**Background:**

Protein aggregation is a hallmark of several neurodegenerative diseases including Huntington's disease and Parkinson's disease. Proteins containing long, homopolymeric stretches of glutamine are especially prone to form aggregates. It has long been known that the small protein modifier, ubiquitin, localizes to these aggregates. In this report, nematode and cell culture models for polyglutamine aggregation are used to investigate the role of the ubiquitin pathway in protein aggregation.

**Results:**

Ubiquitin conjugating enzymes (Ubc's) were identified that affect polyglutamine aggregates in *C. elegans*. Specifically, RNAi knockdown of *ubc-2 *or *ubc-22 *causes a significant increase in the size of aggregates as well as a reduction in aggregate number. In contrast, RNAi of *ubc-1, ubc-13*, or *uev-1 *leads to a reduction of aggregate size and eliminates ubiquitin and proteasome localization to aggregates. In cultured human cells, shRNA knockdown of human homologs of these Ubc's (Ube2A, UbcH5b, and E2-25K) causes similar effects indicating a conserved role for ubiquitination in polyglutamine protein aggregation.

**Conclusion:**

Results of knockdown of different Ubc enzymes indicate that at least two different and opposing ubiquitination events occur during polyglutamine aggregation. The loss of ubiquitin localization after *ubc-1, ubc-13*, or *uev-1 *knockdown suggests that these enzymes might be directly involved in ubiquitination of aggregating proteins.

## Background

In the cell, misfolded proteins are usually degraded. In some cases, when a misfolded protein is not efficiently removed from the cell, protein aggregation will occur. Aggregation occurs in neurodegenerative diseases such as Huntington's disease, Parkinson's disease, Alzheimers disease, amyotrophic lateral sclerosis (ALS), spinobulbar muscular atrophy, dentatorubral-pallidoluysian atrophy, Prion diseases, spinocerebellar ataxias and others [1]. In these diseases, aggregation largely correlates with cell dysfunction and cell death. The role that protein aggregation plays in the damage that occurs in neurodegenerative diseases is not completely clear. It appears that aggregation itself is harmful because the expression of aggregating peptides in transgenic mice can lead to neurodegenerative symptoms [2,3]. However, additional evidence suggests that the aggregates themselves may be protective and that the soluble mutant protein fragments might be toxic [4,5]. Several models have been proposed to explain the toxicity of aggregating proteins including impairments of transcription [6], protein degradation [7], protein trafficking [8], or protein folding [9].

The precise molecular pathway leading to aggregation is not well-known. Structural studies on aggregating polyglutamine proteins indicate that they adopt a β-sheet crystalline conformation characteristic of amyloid fibrils [10]. Aggregation is typically considered to be an unfavorable activity for proteins and, indeed, there are mechanisms to prevent such aggregation. Molecular chaperones seem to prevent the formation of protein aggregates in the cell. Overexpression of chaperone proteins can lead to a decrease in the amount of aggregation [11]. Polyglutamine models in the nematode, *C. elegans*, have indicated that expression of polyglutamine proteins is toxic and that chaperones as well as other proteins can serve to ameliorate that toxicity [12-15].

Early in the study of the protein aggregation phenomenon, it was observed that the small protein, ubiquitin, becomes concentrated at sites of aggregation [16]. Since the initial discovery of ubiquitin in aggregates, it has been found in almost every case of aggregation-related neurodegenerative diseases [1,17]. The nature of the ubiquitin present in the aggregates is unknown. It is generally presumed that ubiquitin is conjugated onto substrate proteins such as the aggregating protein itself or other proteins that colocalize to aggregates. In fact, there is some evidence that aggregating proteins are ubiquitinated [18,19]. However, it is also possible that ubiquitin is present in its monomeric state, or coupled with enzymes of the ubiquitination pathway such as E1, E2, or E3 enzymes.

Proteasomes also localize to protein aggregates in the aforementioned diseases [20]. Although it has been shown numerous times that ubiquitin and proteasomes colocalize to aggregates, the role that either plays in aggregation has not been elucidated. Since aggregated proteins are somewhat resistant to proteolysis [21,22], it is possible that the aggregating proteins are tagged for degradation by ubiquitin, but not degraded, thus concentrating ubiquitin and proteasomes at sites of aggregation. Ubiquitin has many functions in the cell aside from tagging proteins for degradation. It can serve as a modulator of protein trafficking, DNA repair, transcription, etc. [23]. Thus, the role of ubiquitination in protein aggregation need not be restricted to the degradative pathway.

Ubiquitin becomes linked to other proteins via an isopeptide bond between the C-terminal glycine of ubiquitin and a lysine side chain on the modified protein. Ubiquitination occurs via a conserved, three-step process involving the E1, E2 and E3 components of the pathway [24,25]. The E1 ubiquitin activating enzyme first interacts with ubiquitin using an ATP-dependent mechanism to form a thiolester linkage between a cysteine residue on the E1 and the C-terminal glycine of ubiquitin. Ubiquitin is subsequently transferred from the E1 to a cysteine residue on the E2 ubiquitin conjugating enzyme (Ubc). Next, the E3 ubiquitin ligases become involved in the process. The E3s are a diverse class of proteins and they can function in the pathway in one of two ways. Some E3s, such as the HECT domain E3s, form a thiolester linkage with ubiquitin and then subsequently transfer ubiquitin onto the target protein. In contrast, the more abundant E3s, such as the RING domain E3s, do not act as true enzymes, but rather function as a scaffold, interacting with both the target protein and the Ubc, thus allowing transfer of ubiquitin from the Ubc onto the target protein. Ubiquitination can occur in the form of monoubiquitination (addition of a single ubiquitin) or polyubiquitination (addition of a chain of ubiquitins).

In order to investigate the role of ubiquitination in protein aggregation, we have used a transgenic nematode system as well as cultured human cells. The transgenic worm strain expresses GFP fused to a stretch of 82 glutamines (Q82:GFP). This strain of nematodes has large fluorescent aggregates in the body wall muscles of larvae and adults [12]. A similar strain was used to screen for genes that affect the timing of aggregate accumulation [26]. That screen identified the ubiquitin activating enzyme (*uba-1*) as an important polyglutamine regulator. Our results show that perturbation of ubiquitin conjugating enzymes via RNAi has a significant effect on aggregate size and number. Specifically, in nematodes, RNAi of the *ubc-2 *or *ubc-22 *genes reduces the number but increases the size of aggregates. RNAi of *ubc-1 *or ubiquitin itself increases the number but decreases the size of aggregates. In HEK293 cultured cells, shRNA knockdown of the human homologs of these Ubc genes shows similar effects on aggregates, indicating a conserved function for these enzymes. Because there are two different phenoptyes observed upon knockdown of different Ubc enzymes, this suggests that there are at least two separate ubiquitination events that occur in the formation or metabolism of aggregates.

## Results

The RNAi technique was used to knockdown ubiquitination enzymes in cells expressing polyglutamine proteins. The nematode, *C. elegans*, has 22 different Ubc's and 4 Uev's (ubiquitin conjugating enzyme variants) encoded in its genome [27]. Using available cDNA clones for eleven of the Ubc's and two of the Uev's, we performed an RNAi screen of these enzymes.

### A nematode model system indicates Ubc's are involved in protein aggregation

A worm model for polyglutamine protein aggregation was developed by Saytal et al. [12] and later converted into an integrated strain as described [15]. This strain of worms expresses GFP that has been fused to 82 glutamines. The *unc-54 *promoter is used to drive expression of this fusion protein, resulting in expression in body wall muscle cells of the worm. We refer to this strain of worms as the Q82:GFP strain.

**Figure 1:**
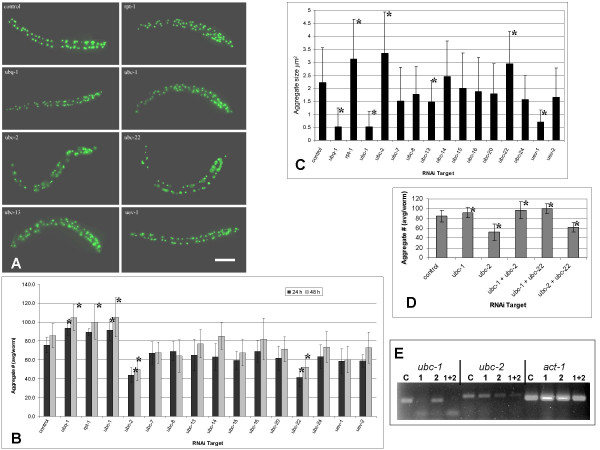
**RNAi of Ubiquitin Conjugating Enzymes in *C. elegans***. The Q82:GFP strain of *C. elegans *expresses polyglutamine aggregates in body wall muscle cells. Synchronized worms from the Q82:GFP strain were treated with RNAi for 48 hours until they reached approximately the L4 larval stage. RNAi treatments included the knockdown of ubiquitin (*ubq-1*), a subunit of the proteasome (*rpt-1*) and 11 different ubiquitin conjugating enzymes. Worms continue to express aggregates after these RNAi treatments, however, significant alterations in numbers and sizes of aggregates occur upon knockdown of specific Ubc's. A) Fluorescent images of control and RNAi treated worms showing the Q82:GFP aggregates. Scale bar is 100 μm. B) The average number of aggregates was counted manually under 40× magnification. RNAi knockdown of ubiquitin (*ubq-1*), proteasome (*rpt-1*) or *ubc-1 *resulted in an increase in the number of aggregates whereas RNAi of either *ubc-2 *or *ubc-22 *showed a significant decrease in the number of aggregates. (* indicates statistical significance at p < .05). C) The average size of aggregates was determined following knockdown of each Ubc after 48 hours of RNAi treatment. The control was found to have an average aggregate area of 2.23 μm^2^. Knockdown of *ubq-1, ubc-1*, or *uev-1 *resulted in a significant decrease in aggregate sizes. Knockdown of *rpt-1*, *ubc-2 *or *ubc-22 *resulted in a significant increase in aggregate sizes. (* indicates statistical significance at p < .05). D) Same procedure as in A except that bacterial feeding cultures were combined to achieve knockdown of more than one Ubc. The *ubc-1 *phenotype is epistatic to both *ubc-2 *and *ubc-22*. There is no additive effect between *ubc-2 *and *ubc-22*. (* indicates statistical significance at p < .05). E) RT-PCR of RNAi treated worms confirms RNAi knockdown. Primers for *ubc-1, ubc-2*, and *act-1 *were used to test RNA levels after RNAi treatments. For each set of primers, PCR template for lane **C **is cDNA from control (pL4440) worms, lane **1 ***ubc-1(RNAi*), lane **2 ***ubc-2(RNAi*), and lane **1+2 ***ubc-1 + ubc-2(RNAi*).

Worms from the Q82:GFP strain were subjected to RNAi of various Ubc's as well as ubiquitin (*ubq-1*) and a proteasome subunit (*rpt-1*). Following RNAi, worms were fixed and analyzed with respect to the number and size of GFP aggregates. Figure 1 shows the results of the RNAi treatments. In these experiments, embryos were collected from gravid adults and then placed directly onto RNAi feeding plates. Thus, exposure to the dsRNA began as soon as these L1 larvae hatched from the egg shell.

RNAi of most Ubc's did not cause a discernable difference in aggregate size or number, however, Figure 1 shows that knockdown of *ubc-2 *and *ubc-22 *resulted in a significant reduction in the number of aggregates per worm and an increase in the average size of aggregates. Knockdown of *ubc-1, ubc-13, uev-1*, and *ubq-1 *resulted in a reduction in the size of aggregates. RNAi of *ubc-1 *and *ubq-1 *also caused an increase in the number of aggregates. No other phenotypes in these worms were noticed besides a marked growth arrest and immobility in the *ubq-1 *and *rpt-1 *RNAi worms after 48 hours.

Because *ubc-1 *showed an opposite phenotype from *ubc-2 *and *ubc-22*, this suggests that there are at least two different ubiquitination events and that these different ubiquitinations may have separate effects on the formation or metabolism of the aggregates. RNAi of *ubq-1*, a gene encoding a polyprotein consisting of 11 ubiquitin peptides, was used to knockdown ubiquitin levels. Although the phenotype with *ubq-1 *RNAi is severe [28], it may not completely eliminate ubiquitin as there is a second gene, *ubq-2*, which also encodes ubiquitin. RNAi knockdown of *ubq-1 *showed a phenotype similar to that of *ubc-1*, suggesting that the UBC-1 mediated ubiquitination event is more prevalent or proceeds ubiquitination by UBC-2 or UBC-22.

Further evidence for the epistasis of *ubc-1 *came from combined RNAi experiments. When *ubc-1 *was knocked down in combination with *ubc-2*, or *ubc-22*, the *ubc-1 *phenotype was observed (Figure 1D). RT-PCR confirmed knockdown of both *ubc-1 *and *ubc-2 *in the combined RNA treatments (Figure 1E). The results from the combined RNAi experiments are consistent with a model where *ubc-1 *functions first in the cascade leading to ubiquitination of aggregating proteins.

### The Ubc phenotype does not affect the solubility or amount of Q82:GFP

There are several possible scenarios to explain how knockdown of Ubc's might affect the size and number of aggregates. First, the resulting Ubc deficiency could affect the total amount of Q82:GFP protein in the cell. Second, the solubility of Q82:GFP could be altered. Third, the composition or density of the aggregate could be affected.

**Figure 2:**
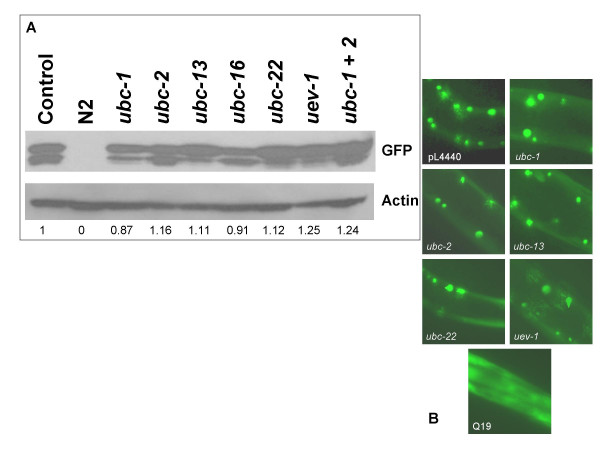
**RNAi treatments do not affect Q82:GFP solubility or overall levels**. The solubility and amount of Q82:GFP proteins were tested after RNAi treatment. A) Levels of Q82:GFP protein were analyzed with western blots using an antibody to GFP. The antibody detects GFP in transgenic strains but not in the wild type, N2, strain. The total levels of GFP (both bands of the doublet) were quantified using densitometry and adjusted based on the relative levels of actin as ascertained by reprobing the blot with anti-actin. The relative levels of GFP based on that analysis are given at the bottom. B) Worms were assessed for changes in Q82:GFP solubility after RNAi treatment for 24 hours. All worms, including the control, have a low level of soluble GFP that is apparent. Aggregates appear as bright green foci. No obvious differences in solubility are apparent between RNAi treatment and the control. The Q19:GFP strain is used to show the distribution of soluble GFP protein.

In order to test the first of these three possibilities, we examined levels of Q82:GFP using western blot analysis (Figure 2A). GFP is detected as a doublet that migrates in the gel to a molecular weight of approximately 33–36 kDa. This weight agrees with the predicted weight of the Q82:GFP fusion protein (37 kDa). The nature of the difference between the two bands in the doublet is unknown. However, there does appear to be some variation in the relative abundance of the two species in some RNAi samples. Future studies will address the significance of these differences. The difference in molecular weight between the two species is too small to be explained by differences in ubiquitination and is more likely to be the result of a smaller modification such as phosphorylation. Quantitative analysis of the levels compared to an actin control show that the absolute levels of Q82:GFP are not significantly altered by the RNAi treatments. Therefore, the differences in aggregate size and number that are seen in RNAi of *ubc-1, ubc-2, ubc-22, ubc-13*, and *uev-1 *are most likely not explained by changes in the expression or stability of the Q82:GFP protein.

Differences in aggregate size and number could also be explained by changes in the solubility of the polyglutamine proteins. This has been observed in several instances. For example, overexpression of chaperone proteins can increase the solubility of polyglutamine proteins and reduce aggregation [15,29]. As a simple test for solubility, we analyzed the amount of soluble GFP in images of live worms, either RNAi treated or control (Figure 2B). Some soluble GFP is observed in Q82:GFP worms at this stage. However, there is no apparent change in the solubility upon any of the RNAi treatments. In contrast, examination of a strain containing a stretch of only 19 glutamines fused to GFP (Q19:GFP) clearly shows soluble GFP (diffuse green fluorescence).

### Knockdown of *ubc-1*, *ubc-13*, or *uev-1 *affects localization of ubiquitin and proteasome to aggregates

**Figure 3:**
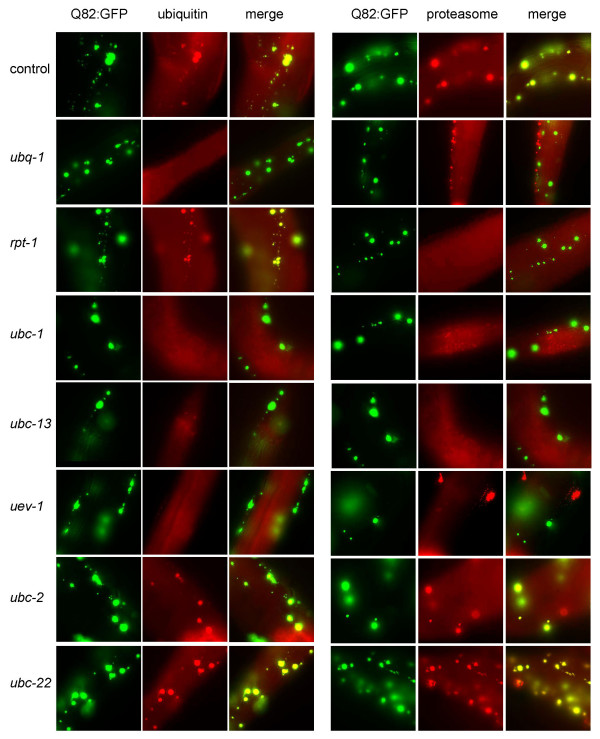
**Ubiquitin and proteasome colocalization to aggregates is affected by Ubc knockdown**. Antibodies to ubiquitin and a proteasome subunit were used to test the effects of Ubc RNAi. Yellow color in the Merge columns indicates colocalization with aggregates. In control worms, ubiquitin and proteasomes colocalize to polyglutamine aggregates (top row). RNAi with *ubq-1 *eliminates localization of both ubiquitin and proteasomes. RNAi with *rpt-1 *does not disrupt localization of ubiquitin to aggregates, but does remove proteasomes, as expected. RNAi with either *ubc-1, ubc-13*, or *uev-1 *diminishes localization of ubiquitin and proteasomes to aggregates, whereas *ubc-2 *and *ubc-22 *show no effect on colocalization.

The knockdown of certain Ubc's has an effect upon aggregate size or numbers. Therefore, we wanted to test whether ubiquitin localization to aggregates is affected. Worms were subjected to RNAi, then, fixed and probed with antibodies to either ubiquitin or a proteasome subunit. Figure 3 shows the results of the colocalization experiments. Staining of the control worms shows that both ubiquitin and proteasome are localized to aggregates. Knockdown of ubiquitin results in the removal of ubiquitin as well as proteasomes from aggregates. This result suggests that ubiquitin localization to aggregates may be required for proteasome localization. Proteasome localization, however, is not required for localization of ubiquitin to aggregates since ubiquitin still localizes to aggregates after *rpt-1 *RNAi.

RNAi of *ubc-1, ubc-13 *and *uev-1 *all resulted in smaller aggregates. Interestingly, these RNAi treatments also showed decreased localization of ubiquitin to aggregates. In addition, proteasome localization was abrogated in these knockdowns (Figure 3), consistent with the hypothesis that ubiquitin localization is a prerequisite for proteasome localization. Loss of ubiquitin from aggregates after knockdown of *ubc-1, ubc-13 *or *uev-1 *cannot be explained solely by the decrease in size since even smaller aggregates in control worms also stain positively with the ubiquitin and proteasome antibodies (Figure 3, top row).

Knockdown of *ubc-2 *or *ubc-22*, which both showed a dramatic increase in aggregate size, shows no effect on localization of ubiquitin or proteasome. Since ubiquitin localization is unaffected by RNAi of *ubc-*2 or *ubc-22*, it is less likely that either of these Ubc's is involved in directly ubiquitinating aggregate proteins. *ubc-1, ubc-13*, and *uev-1 *are more likely candidates for enzymes involved in ubiquitinating aggregate proteins.

### Human Ubc's have a similar affect on polyglutamine aggregation

Our results in *C. elegans *suggest that ubiquitination plays an important role in the aggregation pathway. In order to investigate whether this might also be true in human cells, we performed shRNA experiments in HEK293 cells.

The worm genes showing the most dramatic phenotypes included *ubc-1, ubc-2 *and *ubc-22*. We searched for human orthologs of these Ubc's and identified two very close orthologs of *ubc-1 *and *ubc-2*: Ube2A and UbcH5b, respectively. The human and worm enzymes are extremely similar with Ube2A and UBC-1 showing 84% identity and UbcH5b and UBC-2 having 94% identity. There was no close ortholog for *ubc-22*, however E2-25K (also known as HIP2) was the closest human enzyme with 32% identity. E2-25K is a well studied Ubc enzyme and many activities have been attributed to it such as polyubiquitin chain synthesis, cyclization of ubiquitin chains, neddylation, NF-κB processing, and interaction with Huntingtin [30-33]. Although E2-25K and UBC-22 show a relatively low level of identity, they both contain a C-terminal UBA or ubiquitin associated domain as does Ubc1 of yeast.

**Figure 4:**
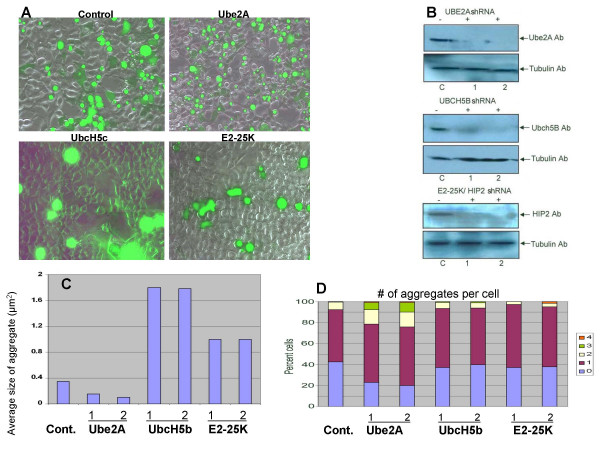
**Ubc knockdown affects polyglutamine aggregation in HEK293 cells**. Knockdown of ubiquitin conjugating enzymes in HEK293 cells was achieved using short hairpin RNA plasmids (shRNA). HEK293 cells were transfected with Q81:YFP plasmid plus either control or Ubc shRNA plasmids. Two different shRNA constructs were used for each Ubc (designated as **1 **and **2 **in panels B-D). Cells were allowed to grow for 72 hrs after transfection. A) Following incubation, RNAi silenced cells were imaged by fluorescence microscopy. The pictures shown are an overlay between a bright field and a fluorescent image. B) Western blotting was performed to detect the level of knockdown of each Ubc using the antibodies indicated. All shRNA constructs achieved significant knockdown of their cognate Ubc. C) Aggregate size was measured after Ubc RNAi. Ube2A knockdown leads to smaller Q81:YFP aggregates however, UbcH5b and E2-25K knockdown increase the size of aggregates. At least 150 aggregates were measured for each shRNA transfection. These data represent the average sizes of aggregates from three independent experiments (> 450 aggregates). D) The number of aggregates per cell after Ubc RNAi was determined by analyzing photomicrographs of transfected cells. In control cells, approximately 42% of cells contain only soluble GFP and no aggregates. Ube2A knockdown causes a higher percentage of cells to form aggregates and also results in an increase in the percentage of cells with more than one aggregate. Knockdown of UbcH5b or E2-25K does not alter the ratio of cells containing aggregates versus cells with soluble GFP. Nor do they significantly affect the average number of cells per aggregate. Data were obtained from three independent experiments, counting 150 cells each time.

shRNA plasmids for the human orthologs were used to knockdown Ubc levels in HEK293 cells. Two different shRNA constructs were used for each gene. shRNA plasmids were transfected together with a plasmid expressing Q81:YFP. Control and shRNA treated cells are shown in Figure 4A. Knockdown of the Ubc's was confirmed by western blot (Figure 4B).

After transfection, cells were examined for the size of aggregates as well as the number of aggregates per cell. Knockdown of Ube2A, the UBC-1 homolog, resulted in a decrease in the average size of aggregates (Figure 4C). In addition, more cells overall had aggregates and a greater percentage of cells had multiple aggregates (Figure 4D). These results are consistent with the effect of *ubc-1 *RNAi in worms. Therefore, the role that this enzyme plays in aggregate formation or metabolism is conserved.

Knockdown of UbcH5b, the UBC-2 homolog, resulted in a significant increase in the size of aggregates but did not affect the number of aggregates per cell (Figure 4C &4D). E2-25K (HIP2) is the Ubc that showed the lowest homology with its worm counterpart, UBC-22. E2-25K showed a modest affect on aggregate size and no affect on numbers of aggregates per cell. The RNAi phenotypes of UbcH5b and E2-25K suggest that any ubiquitination event(s) mediated by these enzymes somehow limits the size of aggregates.

### Human Ube2A and UbcH5b localize to aggregates

**Figure 5:**
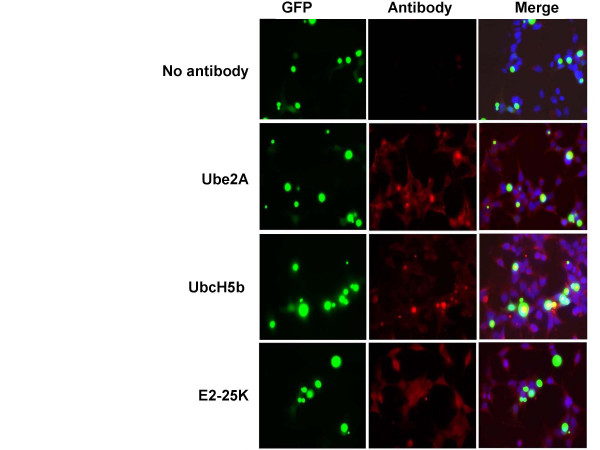
**Ube2A and UbcH5b localize to aggregates**. The localization of Ubc's in cells containing polyglutamine aggregates was determined by immunofluorescence. HEK293 cells were cultured in 24 well plates, fixed and immunostained with antibodies against Ube2A, UbcH5b and E2-25K proteins. Control slides lacked primary antibody. Approximately 150 cells per Ubc enzyme were observed. To quantify colocalization, the percent of cells which contained YFP aggregates that also stained with the Ubc antibody was determined. In Ube2A and UbcH5b there was partial localization (49% and 71% respectively) whereas E2-25K showed no distinct colocalization with aggregates.

Antibodies to the human Ubc's were used to probe cells expressing Q81:YFP. Figure 5 shows the localization of the Ube2A, UbcH5b and E2-25K in HEK293 cells with polyglutamine aggregates. Ube2A and UbcH5b both show strong colocalization to aggregates, whereas E2-25K does not colocalize.

Ube2A is a closely related human ortholog of the worm protein UBC-1. Although we do not know the subcellular localization of UBC-1 in worms, we have shown that UBC-1 affects ubiquitin localization to aggregates. Thus, it is not surprising to find that Ube2A localizes to aggregates. On the other hand, UBC-2, the worm ortholog of UbcH5b, did not affect ubiquitin localization. Therefore, it is curious that it would localize to aggregates. Perhaps it is involved in a minor ubiquitination event on aggregate proteins. Alternatively, UBC-2 might localize to aggregates due to the high concentration of ubiquitin and proteasomes that are present there.

## Discussion

It has long been known that the small protein modifier, ubiquitin, localizes to the large protein aggregates that form in several types of neurodegenerative diseases. We have investigated the role that ubiquitin plays in the formation or metabolism of these aggregates by performing knockdown of Ubc enzymes in the ubiquitination pathway. Our results indicate that ubiquitination plays an important role in polyglutamine protein aggregation.

### Ubiquitination may have multiple roles in protein aggregation

**Figure 6:**
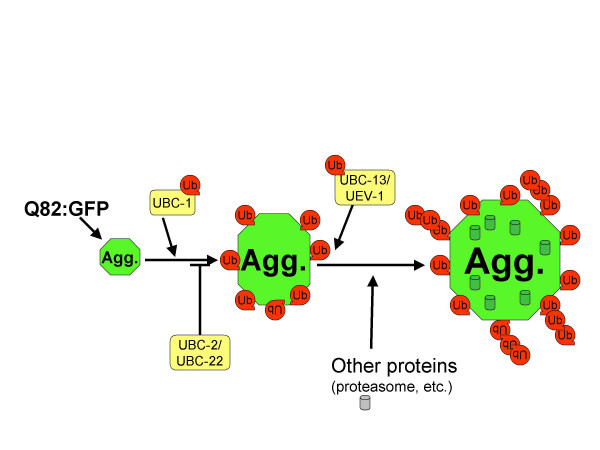
**A model for the role of Ubc's in the formation of polyglutamine aggregates**. In our experimental system, Ubc's are required for polyglutamine aggregates to achieve their normal size. Other research has indicated that aggregates initiate as small particles of aggregating protein that then travel along microtubules to ultimately collect and form large aggregates [42]. Since the knockdown of *ubc-1 *results in smaller, more numerous aggregates, ubiquitination by *ubc-1 *may be needed for small aggregates to come together and form large aggregates. UBC-2 and UBC-22 may normally have an inhibitory role in this process because their knockdown results in larger and less numerous aggregates. Ubiquitination by *ubc-13 *and *uev-1 *may be required to recruit other proteins, such as proteasomes, to the aggregates, thereby allowing aggregates to further increase in size.

Knockdown of Ubc's caused changes in the size or number of polyglutamine protein aggregates. Our results indicate that there are, at minimum, two different ubiquitination events that affect aggregates. This conclusion is made because there are two groups of phenotypes with opposite characteristics: one set of knockdowns showing larger and less numerous aggregates, and one set showing smaller aggregates. Therefore, these two proposed ubiquitination pathways appear to have opposing affects on aggregation. A model for how the Ubc's might be involved in protein aggregation is presented in Figure 6.

Both *ubc-2 *and *ubc-*22 had a similar phenotype: reduction of aggregate number and increase in aggregate size. This indicates that these two ubiquitinating enzymes may be involved in a common process that limits the size of aggregates. It is possible that ubiquitination by *ubc-2 *and *ubc-22 *is important for degrading aggregating proteins or for ridding the cell of aggregates that reach a certain size. However, removal of aggregates does not seem a likely mechanism for *ubc-2 *and *ubc-*22, since their knockdown results in fewer aggregates than normal. Therefore, *ubc-2 *and *ubc-*22 may be involved in limiting the coalescence of smaller aggregates into larger aggregates.

Since they have the same knockdown phenotype and the combined knockdown is not additive (Figure 1D), the two enzymes, UBC-2 and UBC-22, might participate in the same ubiquitination pathway or might function as a dimer. There is evidence from both *in vitro *and *in vivo *studies suggesting the existence of Ubc dimers and Ubc-Uev dimers [34,35]. One interesting aspect of UBC-22 is that it has a non-canonical catalytic site with an awkwardly placed cysteine residue in a context lacking all consensus residues [36]. Thus, it is possible that UBC-22 may not be catalytically active as a Ubc enzyme but rather partners with another functioning Ubc.

*ubc-1, ubc-13*, and *uev-1 *all showed a reduction in the size of aggregates. This reduction in size came without a reduction in the overall levels or a change in solubility of Q82:GFP protein. RNAi of *ubc-1 *had the most dramatic effect on size and also caused aggregates to be more numerous. UBC-1 could be involved in the process of smaller aggregates joining together into larger aggregates (Figure 6).

Since RNAi of *ubc-1, ubc-13 *and *uev-1 *all cause a reduction in aggregate size and loss of ubiquitin from aggregates, it is tempting to speculate that these enzymes might also function in the same pathway. In fact, it has been shown that the yeast homologs of these three enzymes function together in ubiquitination of PCNA. Rad6, the yeast homolog of *ubc-1*, is responsible for adding the initial ubiquitin molecule onto PCNA, and the ubiquitin chain is subsequently extended by the Ubc13/Uev1 dimer [37].

Knockdown of *ubc-1, ubc-13 *or *uev-1 *could cause aggregates to be smaller due to a decrease in overall bulk of the aggregate. Along with many other proteins, it is known that the proteasomes localize to aggregates and, thereby, could contribute to aggregate volume. Proteasome localization is disrupted after RNAi of *ubc-1, ubc-13*, or *uev-1*. Therefore, one possible scenario is that ubiquitination of Q82:GFP or some other protein attracts proteasomes to the aggregate. In the absence of that ubiquitination, proteasomes or other ubiquitin binding structures do not colocalize and thus the overall size of the aggregate is reduced.

### The ubiquitination pathway is conserved

We have used the nematode as a model for understanding the aggregation of polyglutamine proteins. The validity of this model was tested by examining the effects of Ubc RNAi in human cells expressing polyglutamine aggregates. Our results indicate that both worm cells and human cells deal with polyglutamine proteins using a conserved mechanism.

Using shRNA plasmids, we achieved knockdown of three human Ubc's that have homologs in our group of worm Ubc's with strong effects on aggregates. The human Ubc's with strong similarity to the worm enzymes showed phenotypes that mimicked the RNAi phenotype in worms. E2-25K, which has only weak identity to worm UBC-22, showed a less pronounced aggregate phenotype.

## Conclusion

Using model systems, we have shown that knockdown of certain Ubc's can lead to changes in polyglutamine aggregates either in nematodes or human cells. However, the mechanism whereby ubiquitination affects protein aggregation remains unknown and could be either a direct or indirect affect of Ubc knockdown. The result that knockdown of *ubc-1, ubc-13*, and *uev-1 *reduce ubiquitin localization to aggregates favors a model where these enzymes participate directly in ubiquitinating components of the aggregate. It will be important to learn the targets for ubiquitination and to determine which Ubc's and ubiquitin ligases are involved in ubiquitinating those targets.

## Methods

### Worm strains and maintenance

Worms were maintained at 20°C on nematode growth media with OP50 *E. coli *according to standard methods [38]. The integrated Q82:GFP nematode strain is described elsewhere [15]. The OP50 and HT115 bacterial strains as well as N2 worms were obtained from the Caenorhabditis Genetics Center.

### Plasmid constructions

The GATEWAY system (Invitrogen, Inc.) and the vector pL4440GTWY [39] were used to generate RNAi feeding plasmids [40]. RNAi clones for *ubc-1, ubc-2, ubc-7, ubc-8, ubc-13, ubc-14, ubc-16, ubc-20, ubc-22, ubc-24, uev-1, uev-2, ubq-1*, and *rpt-1 *were made using recombinational cloning with pL4440GTWY and entry clones obtained from Mark Vidal and Open Biosystems, Inc. [41].

For aggregate expression in HEK293 cells, the Q81-YFP plasmid was generously provided by Dr James R. Burke (Duke University Medical Center Durham, NC). Expression Arrest™ human shRNA plasmids were from Open Biosystems. Expression Arrest™ contained either the non-silencing sequence which has no homology in mammals (control) or sequences specifically targeting Ube2A (1, RHS1764-9391619; 2, RHS1764-9690190), UbcH5b (1, RHS1764-9392787; 2, RHS1764-9685283), or E2-25K (1, RHS1764-9700185; 2, RHS1764-9100963).

### Nematode RNAi

RNAi was performed as previously described [39]. Plasmids described above were transformed into HT115 *E. coli*. 35 mm NGM plates were supplemented with 1 mM isopropyl-β-thiogalactopyranoside (IPTG) and 100 mg/ml ampicillin and then seeded with 125 μl of saturated overnight culture. Q82:GFP nematode populations were synchronized by using a hypochlorite treatment to yield embryos. Embryos were placed on RNAi-seeded plates 2–4 hours after seeding. Worms were removed from plates after 24 or 48 hours for analysis. Control RNAi was performed using the pL4440 empty vector in HT115.

### Nematode aggregate analysis

Nematodes were fixed in methanol onto polylysine-coated slides and mounted in Vectashield plus DAPI (4,6-diamidino-2-phenylindole). Aggresomes were counted by observation on a Nikon E600 microscope equipped with a QiCam digital camera (Q Imaging). A minimum of 10 worms were analyzed for each condition. Sizes of individual aggregates were determined using Image Pro software. At least 100 aggregates were sized for each RNAi condition. Soluble GFP was observed by anesthetizing nematodes with 25 mg/mL tetramisole (Sigma) in PBS.

Colocalization of ubiquitin and proteasome to aggregates was determined using mouse-anti-ubiquitin (1:200; P4D1 from Santa Cruz Biotechnology) and rabbit-anti-proteasome (1:200; ab2943 from Abcam). Nematodes were fixed with methanol onto polylysine-coated slides and incubated in primary antibody overnight followed by incubation with secondary antibody for 2 hours. Secondary antibodies were goat-anti-mouse Rhodamine and goat-anti-rabbit Rhodamine (Jackson ImmunoResearch) Worms were observed and analyzed using the microscopy tools mentioned above.

### Cell culture maintenance and RNA interference in HEK293 cells

Cell culture reagents were purchased from Mediatech. HEK293 cells were transiently transfected using Arrest-In™ transfection reagent (Open Biosystems) following the manufacturer's protocol. Cell culture was done in DMEM with 10% FBS in an incubator with 5% CO_2_.

shRNA plasmids were isolated using a Qiagen kit and verified using restriction digests. Transfections were done in 6 well plates. For each well, 2 μg of shRNA plasmid and 100 ng of Q81:YFP in 50 μl of serum free DMEM was added to 50 μl of diluted Arrest-In™ and left at room temperature for 15 min. The transfection mixture was added drop wise to the wells and incubated at 37°C; 5% CO_2_. Media was changed 1 day following transfection. Cells were analyzed 72 hrs after transfection.

### Aggregate analysis in HEK293 cells

Images were taken of cells containing fluorescent aggregates 72 hrs after transfection with shRNA plasmids. Each experiment was repeated three times and each time 150 transfected cells were examined. The number of aggregates per cell was counted, and area of each aggregate was measured using SPOT software (Diagnostics Instruments). Area of aggregates was converted to μm^2 ^by calibrating with a micrometer (1 μm was equivalent to 39.6 pixels).

For localization of Ubc proteins in aggregate-containing cells, HEK293 cells were cultured in 24 well plates and transfected with 100 ng of Q81:YFP in each well. Cells were incubated for 48 hrs, then trypsinized and transferred to 4-chambered slides (NUNC). After another 24 hrs, cells were washed once with PBS and fixed with 4% paraformaldehyde (in PBS) at 4°C for 30 min. Cells were checked for fixation under a light microscope. After fixation, cells were washed three times with Tris buffered saline (TBS; 50 mM Tris, 150 mM NaCl pH 7.6) followed by blocking (1% BSA, 0.3% Triton-X-100 in TBS) for 1 hr at room temperature. Primary antibody (rabbit-anti-E2-25K from Boston Biochem 1: 200 or goat-anti-Ube2A polyclonal 1: 100 or goat anti-Ubch5B polyclonal 1:100 both from Santa Cruz Biotechnology) was added in 1% BSA, 0.3% Triton-X-100 and incubated overnight at 4°C. Slides were then washed three times with TBS followed by incubation with secondary antibody (goat-anti-rabbit Rhodamine from Jackson ImmunoResearch or rabbit-anti-goat Rhodamine from Open Biosystems) in 1% BSA, 0.3% Triton-X-100 and incubated for 6 hrs at 4°C in the dark. Cells were washed three times with TBS. Chambers were removed from the slides very carefully until no gum was left on the slide. Prolong Gold Antifade reagent with DAPI (Molecular Probes) was added and was allowed to cure overnight at 4°C in dark. Cells were examined using a Leica DMIRB inverted microscope equipped with a SPOT RT slider color digital camera. Images were obtained with blue, green, red fluorescence filters. Images were processed by C IMAGING software (SIMPLE PCI Inc.).

### RT-PCR

Worms were grown on RNAi plates for 48 hours, collected by centrifugation, washed 5 times with PBS, and frozen at -80°C. Frozen pellets were ground using a mortar and pestle in liquid N_2_. RNA extractions were performed using the QiaShredder and RNeasy miniprep kits (Qiagen). cDNA was synthesized from the RNA samples with the Transcriptor First Strand cDNA kit (Roche) using 10 μl of RNA as template. PCR reactions used 2 μl of cDNA and the following primers: **UBC1F **CAAATAAACCGCCAACCGTCAA, **UBC1R **TCTCATATTCCCGTCGATTTTCTT, **UBC2F **ACTTCCCAACAGACTATCCATTCA, **UBC2R **AGCGTACTTTTGCGTCCATTCTCT, **ACT1F **CCGCCGGAATCCACGAGACT, **ACT1R **GTGGAGAGGGAAGCGAGGATAGAT.

### Immunoblotting

#### Nematodes

Worms were grown on RNAi plates for 48 hours, collected by centrifugation, washed with sterile water, and suspended in 1× protein sample buffer with BME. Samples were heated at 100°C for 5 minutes, resolved on 10% SDS-polyacrylamide gels, transferred to PVDF membranes, and incubated overnight with mouse anti-GFP antibody (1:200; Santa Cruz Biotechnology) at 4°C and 1 hour at room temperature with secondary antibody (1:10,000, Pierce) Blots were reprobed with anti-actin antibody (1:500; C4 from Abcam) Bound antibody was detected with SuperSignal West Pico Chemiluminescent Substrate (Pierce). Densitometric measurements were performed using Image J software.

#### HEK293 cells

Cells were collected from plates following trypsinization, then washed with 1× PBS and suspended in PBS. Lysis was done by boiling in loading buffer (50 mM Tris-HCl, pH 6.8 10% SDS, 0.1% bromophenol blue, 10% glycerol) followed by 1 min sonication. Lysates were centrifuged at 14,000 rpm for 10 min at 4°C. Samples were electrophoresed through 10% SDS-polyacrylamide gels and transferred to PVDF membranes. Membranes were probed overnight at 4°C with using the same primary antibodies used for immunofluorescence (dilutions: anti-E2-25K 1:1000, anti-Ube2A 1:200, anti- UbcH5b 1:200, anti-tubulin from the Developmental Studies Hybridoma Bank at the University of Iowa 1:1000). The secondary antibody (goat-anti-mouse HRP or rabbit-anti-goat HRP; Pierce) used diluted 1:10,000 and bound antibody was detected with SuperSignal West Pico Chemiluminescent Substrate.

## Authors' contributions

RAH performed sizing of aggregates in nematodes and localization of ubiquitin and proteasome to aggregates in nematodes. PS did all experiments using HEK293 cells. CH determined numbers of aggregates per worm and also did the microscopic solubility analysis. KAC and GAC provided valuable strains and important technical assistance early in the project. RdB and RH assisted with shRNA transfections and immunological analysis in HEK293 cells. LB did the Western blots and RT-PCR as well as provided oversight of the project and did the majority of writing. All authors have read and approved the manuscript.
